# Perinatal outcomes of high dose versus low dose oxytocin regimen used for labor induction and factors associated with adverse perinatal outcome in four hospitals of Ethiopia: a multicenter comparative study

**DOI:** 10.1186/s12884-021-04052-5

**Published:** 2021-08-28

**Authors:** Melese Gezahegn Tesemma, Demisew Amenu Sori, Desta Hiko Gemeda

**Affiliations:** 1 Department of Obstetrics and Gynecology, Arsi University, Asella, Ethiopia; 2 Department of Obstetrics and Gynecology, Jimma University medical center, Jimma, Ethiopia; 3 Department of Epidemiology, Jimma university Institute of health, Jimma, Ethiopia

**Keywords:** Labor induction, Oxytocin regimen, High dose oxytocin, Low dose oxytocin, perinatal outcome, adverse perinatal outcome

## Abstract

**Background:**

There is limited evidence on effect of high and low dose oxytocin used for labor induction on perinatal outcomes. We compared perinatal outcomes among pregnant mothers who received the two different oxytocin regimens and identified risk factors associated with adverse perinatal outcomes.

**Methods:**

Facility based comparative cross-sectional study was conducted in four hospitals of Ethiopia over eight month’s period during 2017/2018 year with 216 pregnant women who received high and low dose oxytocin for labor induction. Socio-demographics, reproductive characteristics of mothers and perinatal outcomes data were collected and entered into Epi-data version 3.1 and then exported to SPSS version 20 for cleaning and analysis. Chi-square test and logistic regression were done to see the effect of different oxytocin regimens on perinatal outcome. The result was presented using 95 % confidence interval of crude and adjusted odds ratios. P-value < 0.05 was used to declare statistical significance.

**Result:**

Higher adverse perinatal outcomes (29 % vs. 13.9 %, p = 0.005) and higher non-reassuring fetal heart rate pattern (23.1 % vs. 7.4 %, p = 0.001) was observed among mothers who received high dose oxytocin compared to mothers who received low dose oxytocin. Using high oxytocin dose [AOR = 2.4, 95 % CI: 1.1, 5.5], caesarean birth [AOR = 9.3, 95 %CI: 3.8, 22.5], instrumental birth [AOR = 7.7, 95 % CI: 2.1, 27.8], and antepartum hemorrhage [AOR = 17.8, 95 %CI: 1.9, 168.7] were risk factors of adverse perinatal outcomes.

**Conclusions:**

There was significance difference in the occurrence of adverse perinatal outcomes among pregnant mothers who received high and low dose of oxytocin. Using high dose oxytocin, antepartum hemorrhage, caesarean birth and instrumental birth were associated with increased risk of adverse perinatal outcomes. We recommend using low dose oxytocin for better perinatal outcomes.

## Background

Labor induction is aimed at achieving vaginal birth when the benefits of giving birth out-weigh the risk of continuing the pregnancy to the mother and perinatal outcome [1, 2]. Oxytocin is synthetic polypeptide hormone that is used to stimulate uterine contractions [2, 3]. Induction of labor (IOL) is associated with poorer perinatal outcomes when compared with spontaneous labor. There is a greater risk of neonatal and fetal complications like fetal heart rate decelerations, low 5-minute Apgar score, admission to a neonatal intensive care unit (NICU) and delayed initiation of breastfeeding [4].

Oxytocin regimen can be either high dose or low dose depending on the starting dose, the amount and rate of sequential increases in the dose of oxytocin [5]. Intervals to increase oxytocin doses vary from 15 to 60 min in different trials [6–9]. Starting dose of high-dose regimes ranged from 4 to 10 mili-Unit/ minute (mU/min) while that of low dose ranges from 1 to 4 mU/min [5]. Even though Ethiopian national guideline and other study recommends use of low dose oxytocin regimen for labor induction ([3], Management Protocol on Selected Obstetric Topics: Federal Democratic Republic Of Ethiopia, Ministry Of Health, unpublished), there are also centers which are utilizing high dose oxytocin regimen one of which is Jimma university medical center (JUMC) (Segni H, Niguse D. et al.: Obstetrics management guideline in JUSH Jimma University, unpublished).

To date, there is no strong recommendation of a particular dosage of oxytocin regimen for labor induction [10–12]. Although there are few studies conducted on outcomes of induction using oxytocin in Ethiopia, there is no evidence that compares the effect of high dose and low dose oxytocin regimen on perinatal outcomes. This study was therefore aimed at determining the effects of oxytocin regimens on perinatal outcomes by comparing high dose and low dose oxytocin regimens.

## Method & Material

### Study area, study period and study design

Facility based comparative cross-sectional study was conducted in four hospitals of Ethiopia namely Jimma University Medical Centre (JUMC), Shanan Gibe general hospital, Arbaminch General Hospital and Kuyu General Hospital over eight months period during 2017/2018 year. JUMC is a tertiary teaching hospital with high birth rate capacity about 4300 births per year and uses high dose oxytocin regimen while the other three were with lower birth rate capacity utilizing low dose oxytocin regimen. Arbaminch general hospital has 1500 births per year, Kuyu general hospital about 1350 births per year and Shenen gibe general hospital has nearly 1400 births per year. The methodology used in the current study is similar to that of another study related to the same project which has been published in BMC Pregnancy and Childbirth [13].

### Study population

Pregnant women with singleton gestations who undergo induction of labor at term and beyond were included. Pregnant mothers with Intra Uterine Fetal Death (IUFD), critically ill pregnant mothers, pregnant mothers with lethal congenital anomaly, pregnancies complicated by cord prolapse, induced pregnancy for whom cesarean section (CS) was done for non-obstetric indication like social reason were excluded from the study[13].

### Sample size & sampling technique

The required sample size was determined by using double population proportion using Epi info 7 software, considering the following parameters: Proportion of non-reassuring fetal heart rate patterns (NRFHRP) among laboring mothers who received high dose of oxytocin (54.16 %) and low dose of oxytocin (34.37 %) [9], 5 % level of significance, power of 80 % and 1:1 ratio of exposed to un-exposed. Thus total sample size will be 196. Adding 10 % for non-response, 216 laboring mothers were recruited for the study. Thus, 108 pregnant women were selected from JUMC while the rest 108 pregnant women were recruited from three general hospitals. All pregnant women who had undergone induction of labor during study period were recruited consecutively using the inclusion criteria.

### Data collection, entry and analysis

Socio-demographics, reproductive characteristics of mothers and perinatal outcomes data were collected and entered into Epi-data version 3.1 and then exported to SPSS version 20 for cleaning and analysis. Chi-square test and logistic regression were done to see the effect of different oxytocin regimens on perinatal outcome. Variables showing association on bivariate analysis whose P-values were < 0.25 were taken to multivariate regression model to control the confounding factors. The result was presented using 95 % confidence interval of crude and adjusted odds ratios. P-value < 0.05 was used to declare statistical significance.

### Ethical considerations

Ethical clearance to conduct the research was obtained from institutional review board of Jimma University and written consent was obtained from study participants. All the information collected from the study participants were handled confidentially by omitting their personal identifiers and the data were used for the research purpose only. Participants were told by the language they understand that they have the right to participate in or withdraw from the study. All methods were performed in accordance with the relevant guidelines and regulations in accordance with Helsinki declarations.

### Operational definitions

In this research, the following operational definitions were used.

#### Non reassuring fetal heart rate patterns

fetal heart rate pattern of either persistent category II or category III unresponsive to conservative measures necessitating immediate delivery.

#### Advanced neonatal resuscitation

a newborn who required supplemental oxygen, sucking meconium from oropharynx and intubation immediately after birth.

#### Low APGAR score

First minute APGAR less than 5 or fifth minute APGAR score of less than 7.

#### Neonatal sepsis

Any infection in the neonate occurring within 28 days of birth.

#### Adverse perinatal outcomes

A neonate with one of the following adverse perinatal outcomes but not limited to: low APGAR score, admission to NICU, meconium at birth, need of advanced resuscitation, neonatal sepsis, early neonatal death.

#### Low dose oxytocin regimen

Initial dose of 2mU/min increased by 2mU/min every 30 min up to a maximum of 40mU/minute [13].

#### High dose oxytocin regimen

Initial dose of 6mU/min increased by 6mU/min every 20 min up to a maximum dose of 92.8 mU/min [13].

## Results

### Socio-demographic characteristics of study participants

We have enrolled two hundred sixteen laboring mothers to this study. The mean age of study participants was 26 years. One fifth (18.5 %, 40/216) of laboring mothers were illiterate. Majority138 (64 %) of the study participants were living in urban area with comparable distribution to the two study groups. Out of 216 participants, 128 (59.3 %)) of inductions were conducted for premature rupture of membrane (PROM) while 49 (22.7 %,) were conducted for hypertensive disorder of pregnancy (HDP). Mean weight of newborns was 3260gm (SD + 449). Surprisingly equal number of male and female neonates was delivered in both groups with male to female ratio of 1.3:1 (Table 1).

**Table 1:** Socio-demographic and reproductive characteristics of the participants in four hospitals, October 1, 2017 to May 30, 2018

Variables	Category	Type of oxytocin regimen		Total No (%) *N* = 216 n (%)
		High dose (*N* = 108) n (%)	Low dose (*N* = 108) n (%)	
Age in years	< = 19	4(3.7)	5(4.6)	9(4.2)
	20–29	76(70.4)	81(75.0)	157(72.7)
	≥ 30	28(25.9)	22(20.4)	50(33.1)
Ethnicity	Oromo	60(55.6)	56(51.9)	116(53.7)
	Amhara	27(25)	8(7.4)	35(16.2)
	Gamo	0(0)	23(21.3)	23(10.6)
	Dawro	5(4.6)	12(11.1)	17(7.9)
	Gurage	9(8.3)	4(3.7)	13(6.0)
	Others	7(6.5)	5(4.6)	12(5.6)
Parity	Nulliparous	56 (51.9)	32 (29.6)	88 (40.7)
	Multipara	52 (48.1)	76 (70.4)	128 (59.3)
Monthly income	In USD	181	151	166
Sex of newborn	Male	61(56.5)	61(56.5)	122(56.5)
	Female	47(43.5)	47(43.5)	94(43.5)

Of all 216 babies born, three neonates has complicated by early neonatal death. Overall, adverse perinatal outcomes were observed among 47 (21.8 %) of study participants. Of these 47 neonates with adverse neonatal outcomes, 32(68 %) were observed among high dose group (HDG) while the remaining 15(32 %) of adverse perinatal outcome were observed among low dose group (LDG). Common adverse perinatal outcomes were NRFHRP, 33 (15.3 %) followed by need for advanced neonatal resuscitation, 20 (9.3 %), thick meconium, 19 (8.8 %) and need of referral to NICU, 16 (7.4 %) (Table 2).

**Table 2:** Cross-tabulation of perinatal outcomes among participants with high dose and low dose oxytocin regimen in four hospitals, October 1, 2017 to May 30, 2018

Perinatal Outcomes	Response	Oxytocin Regimen		*P*- Value
		High Dose (*N* = 108)n (%)	Low Dose (*N* = 108)n (%)	
Adverse neonatal outcome	Yes	32(29.6)	15(13.9)	0.005
	No	76(70.4)	93(86.1)	
Non-reassuring fetal heart rate pattern	Yes	25(23.1)	8(7.4)	0.001
	No	83(76.9)	100(92.6)	
Grade 2 or 3 MSAF* at Birth	Yes	12 (11.1)	7 (6.5)	0.230
	No	96(88.9)	101 (93.5)	
Advanced neonatal resuscitation needed	Yes	14 (13)	6 (5.6)	0.06
	No	94(87)	102(94.4	
Neonate referred to NICU*	Yes	10(9.3)	6 (5.6)	0.299
	No	98 (90.7)	102 (94.4)	
First minute APGAR	APGAR < 5	1 (0.9)	2 (1.9)	1.000*
	APGAR > = 5	107 (99.1)	106 (98.1)	
Fifth minute APGAR	APGAR < 7	1 (0.9)	4 (3.7)	0.369*
	APGAR > = 7	107 (99.1)	104 (96.3)	
Outcome of neonate on discharge	Alive	107 (99.1)	106 (98.1)	1.00*
	Dead (ENND)	1 (0.9)	2 (1.9)	

*Fisher’s Exact Test was used.

On cross-tabulation, occurrence of NRFHRP and composite adverse perinatal outcome has showed significant relation with use of different oxytocin regimens while other outcome variables like thick meconium at birth, need of advanced neonatal resuscitation, need of referral to NICU, first minute APGAR < 5, Fifth minute APGAR < 7, Neonatal life status on discharge showed no stastical relation. Accordingly, prevalence of NRFHRP were 23.1 and 7.4 % (*P* = 0.001) among HDG and LDG respectively while overall prevalence of adverse perinatal outcomes was 29.6 and 13.9 % (*P* = 0.005) respectively (Table 2).

### Factors associated with adverse perinatal outcome

On bivariate logistic regression residence, misoprostol use, and neonatal weight, uterine hyper stimulation did not show any kind of association with adverse perinatal outcome while maternal age < 19 years [COR = 4.4, 95 %CI: 1.0,19.4], oxytocin regimen [COR = 2.6, 95 %CI: 1.3, 5.2], caesarean birth [COR = 9.0, 95 % CI:4.0, 20.6], instrumental birth [COR = 7.8 95 % CI: 2.6, 23.7], unfavorable Bishop score [ COR = 3, 95 % CI:1.3,6.6] presence of adverse maternal outcome [COR = 2.8, 95 % CI: 1.1, 7.1] and APH as indication [COR = 8.8, 95 %CI:(1.3, 57.0)] has showed statistically significant association with occurrence of adverse perinatal outcome at P-Value < 0.05 (Table 3).

**Table 3:** Risk factors associated with poor neonatal outcomes in four hospitals, October 1, 2017 to May 30, 2018

Variables	Category	Adverse neonatal outcomes		COR (95 %CI)	*P*-value	AOR (95 %CI)	*P*-value
		Yes n (%)	No n (%)				
Oxytocin regimen	High dose	32(29.6)	76(70.4)	2.6(1.3, 5.2)	0.006	2.4(1.0, 5.5)	0.039*
	Low dose	15(13.9)	93(86.1)	1		1	
Mode of birth	CS	30(41.7)	42(58.3)	9.0(4.0, 20.6)	0.000	9.4(3.8, 22.8)	0.000*
	Instrumental	8(38.1)	13(61.9)	7.8(2.6, 23.7)	0.000	7.8(2.2, 28.3)	0.002*
	SVD	9(7.3)	114(92.8)	1		1	
Indication for induction	PROM	25(19.5)	103(80.5)	0.9(0.3, 2.3)	0.751	1.0(0.3,3.2)	0.943
	HDP	8(16.3)	41(83.7)	0.7(0.2, 2.2)	0.527	0.6(0.1,2.0)	0.373
	APH	5(71.4)	2(28.6)	8.8(1.3, 57.0)	0.023	17.8(1.9,168.7)	0.012*
	Chorio-amnionitis	3(60)	2(40)	5.3(0.7,39.0)	0.105	9.0(0.8,97.0)	0.071
	Post term	6(22.2)	21 (77.8)	1		1	

However, on multivariate model, only high dose oxytocin regimen [AOR = 2.4, 95 %CI: 1.1, 5.5], Caesarean birth [AOR = 9.3, 95 % CI: 3.8, 22.5], instrumental birth [AOR = 7.7, 95 % CI: 2.1, 27.8], APH as indication for induction [AOR = 17.8, 95 % CI: (1.9, 168.7)] were found to be associated with adverse perinatal outcome at *P*-value < 0.05 (Table 3).

## Discussion

Using high dose oxytocin was associated with increased risk of developing adverse perinatal outcomes by 2.5 times compared to low dose oxytocin regimen. Neonates born from laboring mothers who received high dose of oxytocin were more at risk of developing adverse neonatal outcomes. This finding was inconsistent with other studies that showed no significant difference on perinatal outcomes with regard to oxytocin regimen [5, 7, 9, 11, 12] and one other study that showed decreased risk of neonatal sepsis with high dose oxytocin [14]. The increased risk of adverse perinatal outcome observed in our study can be explained by the fact that high dose oxytocin is associated with uterine hyper-systole [5, 6] and non- reassuring fetal heart rate patterns [12] that in turn negatively affects the perinatal outcomes. In this study it was found that prevalence of NRFHRP were 3.1 times higher among HDG than LDG. NRFHRP, being one element of composite adverse perinatal outcome might have contributed to this increased risk of adverse perinatal outcome among HDG.

Cesarean birth and instrumental birth were also associated with increased risk of developing adverse perinatal outcomes by 9.4 times and 8 times compared to vaginal mode of birth in this study. This is consistent with other studies that showed increased risk of perinatal morbidities [15, 16]. One study showed increased risk of fetal scalp bruises and caput succedaneum with operative vaginal birth compared with spontaneous vaginal birth[15] while other study showed significantly higher rates of intracranial hemorrhage, brachial plexus, facial nerve injury, seizure, low 5-minute Apgar score, assisted ventilation among operative deliveries compared with spontaneous vaginal birth [16]. The other justification might be the indications to do C/S or to apply instrument might have contributed for this increased risk of adverse perinatal outcome. In this study, nearly half of C/S and instrumental birth were performed for non-reassuring fetal heart rate pattern; and 70 % of adverse perinatal outcomes was attributed to non-reassuring fetal heart rate pattern. With regard to C/S, during C/S the fetus may develop some anesthesia related complications.

Similarly, antepartum hemorrhage (APH) as indication of IOL was associated with increased risk of developing poor perinatal outcomes by 18 times compared to post-term indication. The fact that APH chiefly abruption-placenta, causes severe perinatal morbidities, severe neonatal acidaemia & cerebral palsy might have led to poor perinatal outcomes [1]. It is not uncommon to find hypoxia-associated periventricular leukomalacia and sudden infant death syndrome in newborns given birth after placental abruptions [1]. So, presence of APH has something to do with increment of adverse perinatal outcome. We didn’t find any more literature that has similar intent to compare with on this finding. Thus, this finding can be used as a base line data for future studies.

The limitation to this study was, it was conducted in a teaching and public health facilities where complicated pregnancies come to tertiary center JUMC in this case. The parity of the study participants were not matched among the two groups proportion of primi-gravida being higher at high dose center. It is known that primi-gravidity is associated with higher adverse perinatal outcome compared with multiparas. These two factors might have affected the finding.

It was found that oxytocin regimen didn’t show significant association with induction success and adverse maternal outcome in another article of the same project unlike adverse perinatal outcome that increase with high dose oxytocin [13, 17].

## Conclusions

High dose oxytocin regimen, antepartum hemorrhage, caesarean delivery and instrumental delivery were associated with increased risk of developing adverse perinatal outcomes. The study favored use of low dose oxytocin as it’s associated with decreased risk of adverse perinatal outcomes without affecting induction success and maternal outcome.

## Data Availability

The data used to generate and or analyze the current study are available from the corresponding author upon the request.

## Abbreviations

APH: Ante Partum Hemorrhage
AOR: Adjusted odds ratio
ARM: Artificial Rupture of Membranes
CI: Confidence Intervals
CPD: Cephalo Pelvic Disproportion
COR: Crude odds ratio
C/S: Caesarean Section
DPM: Drop Per Minute
HDG: High dose group
IOL: Induction of labor
JUMC: Jimma University Medical Centre
LDG: Low dose group
mu/min: Mili-unit/minute
NRFHRP: Non reassuring fetal heart rate patterns
PROM: Premature rupture of membrane
SPSS: Statistical Package for Social Scientists
